# On the Cause of Malaria

**Published:** 1845-01

**Authors:** Washington L. Atlee

**Affiliations:** Professor of Chemistry in Pennsylvania College


To the Editor of the Medical Examiner.

*Lancaster*, December 23d, 1844.

My Dear Sir.—I replied to your strictures on my Introductory Lecture before the members of my class on the same evening of their publication in the Examiner. To them, of course, a reply was due, and here I intended to have left the matter. But after-reflection has convinced me that the pages of the Examiner are its proper place; because the question of the cause of Malaria not being settled, the observation which has fallen from you, an eminent teacher, and editor of a periodical extensively circulated—may not only have a tendency to stifle inquiry, but reflect injuriously on me for advancing opinions which, you say, have been "every where rejected as inconsistent with undoubted facts." Your very frank and courteous manner in discussing this question at your house, on the 12th inst., leads me to believe that this course will be perfectly acceptable to you, and that you will cheerfully publish this letter, which contains, in substance, with slight additions, the reply I made to your objections then.

As I had not positively stated that sulphuretted hydrogen was the miasmatic agent, of course any argument to the contrary was uncalled for. I did, however, assert, what I now re-assert, that "there *is* a well grounded *supposition* that the injurious effects of malarious districts are dependent upon the presence of this compound gas; and *should this prove to be the case*, it will afford a beautiful explanation of the manner by which chlorine acts the part of a disinfecting agent." (*Introductory Lecture*, p. 11.) Nor am I aware, that "this suggestion,' 'made a few years ago by a distinguished European,' 'was *everywhere* rejected as inconsistent with undoubted facts.'" (*Med. Exam. Dec.* 14, 1844, p. 298.) I would refer you, and your readers, to an able essay, "*On the Active Principles of Malaria*," by Daniel P. Gardner, M. D., Prof. of Chem. in Hampden Sidney College, Virginia, published in the April number, 1843, of the American Journal of Medical Science, p. 279.

Your argument, in reference to the evolution of this gas in large cities, if it prove anything, goes rather to establish the position you are combating. If "the inhabitants of large cities are *continually* breathing an atmosphere *highly charged* with this offensive gas," why is it that the sense of smell is never offended by it? I admit that there are constant sources of sulphuretted hydrogen in large cities, and the fact you adduced in conversation is a sufficient proof of it—that the white paint in the city of Philadelphia is almost immediately tarnished by this gas. This very fact, fortunately, cannot be reasoned away, and proves how sulphuretted hydrogen may act the part of a miasmatic agent in country districts, while it is stripped of its devasting influence in large cities. The metallic bases of the paints on the numerous buildings, act as disinfecting agents. No sooner is sulphuretted hydrogen eliminated, than the paint attacks it, and either forms a sulphuret with its metallic base, or a hydrosulphate with its oxide—thus, in either case, at once destroying its miasmatic character, and protecting the inhabitants of cities both from its malarious influence, and the necessity of respiring and smelling this most "offensive gas." In country districts, however, it is permitted to have full and free scope, unchecked by these disinfecting agents. If, therefore, sulphuretted hydrogen be miasma, any agent which completely changes its character, either by decomposition or through new combinations, must destroy its malarious influence; and, consequently, if "cities are the most exempt from malarious diseases," even where the "atmosphere is highly charged with this offensive gas," it must be because the bane has here its antidote. Besides, other substances are evolved with it in cities, which have a tendency to affect its character.

I am not fond of controversy, nor do I desire to engage now in the discussion of this question. I may, however, at some future time take up its consideration, and endeavour to satisfy my own mind, whether there is any miasmatic agent that has more than a *hypothetical* existence. For the present, I shall consider that *sulphuretted hydrogen* is as well-founded a *supposed* agent as any other.

With regard to medical chemistry: I *know* that a few years ago *medical* chemistry was *not* taught in Philadelphia, and I think it cannot be asserted that it "is now very generally" taught in Medical Colleges. My observations, therefore, on this subject, are not inappropriate, neither are they *personal*, at least I have not made them so. With the views I entertain of chemical teaching to a medical class, I cannot but rejoice in your assurance, that I have the "example as well as countenance" of other and more distinguished men in endeavouring to accomplish a necessary reform, by pursuing a course, which *I had planned for myself*, unconscious of the existence of abler coadjutors.

Hoping that our intercourse, as heretofore, may be frank and friendly,

I remain yours very respectfully,

WASHINGTON L. ATLEE.

To Prof. R. M. Huston.

It is the general practice of Journalists, we believe, to exclude *reclamations*, as the only way to avoid confusion and unpleasant collisions. We have deviated from this rule by the insertion of Professor Atlee's letter, because he thinks our remarks on his Lecture are calculated to "reflect injuriously" on him as a medical teacher; but in doing so it is not our purpose to establish a precedent for our editoral guidance on any future occasion. Nothing was farther from our thoughts than to charge our friend with either personality or unsound teaching—such an idea never crossed our mind. In reference to the point in dispute between us, we are glad to learn from Dr. Atlee, that it is his intention to investigate the "cause of malaria," and shall be still more gratified if his investigations shall lead to anything more positive on the subject than we now possess. Of one thing we are very sure, that he will find in the course of his investigations, plenty of testimony to prove that the malaria which causes intermittent fever, occurs abundantly in situations where there are no unusual sources of sulphuretted hydrogen, and that many persons are exposed, by their daily avocations, to the direct influence of that gas, in a state of considerable concentration, without being at all affected by intermittent, remittent, or any other form of fever. But this is a point which may easily be decided by direct experiment, and we know of no one who can better do it than our correspondent. It is a well established fact, that a few hours exposure, especially after night fall, in a malarious district, is sufficient to induce disease of a particular type. Now let any number of persons be subjected, for the requisite time, to the influence of sulphuretted hydrogen, in as concentrated a state as can well be borne, *without the presence of any of the modifying agents to which Dr. Atlee refers*, and see how many will fall victims to ague. Whatever, however, may be the result of Dr. A.'s investigations on this interesting subject, we shall be glad to be the medium of conveying them to the profession.

